# Crystal structure of high-spin tetra­aqua­bis­(2-chloro­pyrazine-κ*N* ^4^)iron(II) bis­(4-methyl­benzene­sulfonate)

**DOI:** 10.1107/S2056989015010713

**Published:** 2015-06-13

**Authors:** Bohdan O. Golub, Sergii I. Shylin, Sebastian Dechert, Maria L. Malysheva, Il‘ya A. Gural‘skiy

**Affiliations:** a Department of Chemistry, Taras Shevchenko National University of Kyiv, Volodymyrska st. 64/13, Kyiv 01601, Ukraine; b Institute of Inorganic Chemistry, Georg-August-University Göttingen, Tammannstrasse 4, Göttingen D-37077, Germany

**Keywords:** crystal structure, iron(II) complex, 2-chloro­pyrazine, hydrogen bonding, π–π contacts

## Abstract

The title salt, [Fe^II^(C_4_H_3_ClN_2_)_2_(H_2_O)_4_](C_7_H_7_O_3_S)_2_, contains a complex cation with point group symmetry 2/*m*. The high-spin Fe^II^ cation is hexa­coordinated by four symmetry-related water and two *N*-bound 2-chloro­pyrazine mol­ecules in a *trans* arrangement, forming a distorted FeN_2_O_4_ octa­hedron. The three-dimensional supra­molecular structure is supported by inter­molecular O—H⋯O hydrogen bonds between the complex cations and tosyl­ate anions, and additional π–π inter­actions between benzene and pyrazine rings. The methyl H atoms of the tosyl­ate anion are equally disordered over two positions.

## Abstract 2

Between the tosyl­ate anions and the octa­hedral complex cations consisting of Fe^II^, four aqua and two *N*-bound 2-chloro­pyrazine ligands, weak O—H⋯O as well as π–π inter­actions play important roles in the mol­ecular self-assembly, resulting in the formation of a three-dimensional structure.

## Chemical context   

Transition metal complexes containing pyrazine or substituted pyrazines as ligands are of current inter­est due to their supra­molecular arrangements and the probability of being spin-crossover compounds. Spin crossover, sometimes referred to as a spin transition or a spin equilibrium behaviour, is a phenomenon that occurs in some metal complexes wherein the spin state of a compound changes *via* influence of external stimuli such as temperature, pressure, light irradiation, magnetic field or guest effects (Gütlich & Goodwin, 2004 ▸). As a result of the appearance of such features as thermochromic effects, magnetic susceptibility changes, changes of cell volume, *etc*. that accompany the mol­ecular switching between high-spin and low-spin states, they can be applied in the development of micro-thermometers and photonic devices (Gural’skiy *et al.*, 2012 ▸).
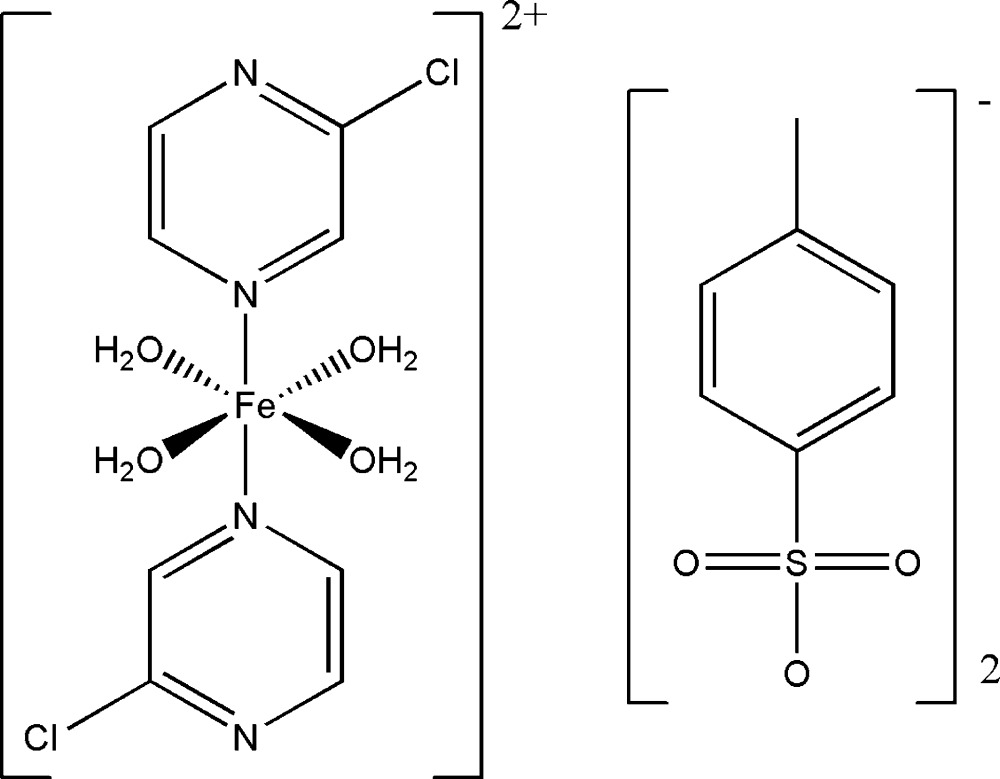


Aromatic ligands bearing two or more N atoms are known for their ability to form different coordination polymers and mol­ecular complexes. Thus, a number of mononuclear high-spin Fe^II^ complexes with substituted pyrazines have been reported recently (Shylin *et al.*, 2015 ▸). These heterocyclic ligands are also known for their ability to create three-dimensional metal-organic framework structures, so called analogues of Hofmann clathrates with general formula {Fe(*L*)_*x*_[*M_y_*(CN)_*z*_]}_∞_ where *M* = Ni, Pd, Pt, *etc*. Series of thio­cyanato coordination polymers [*M*(NCS)_2_ *L*_2_]_∞_ (with *M* = Mn, Fe, Co, Ni, and *L* = pyrazine) in which the small-sized thiocyanate anions are terminally N-bound and therefore not involved in any magnetic exchange interactions are also known (Wriedt & Näther, 2011 ▸). Although 2-chloro­pyrazine could possess a *N,N′*-manner of coordination, it is frequently found to act as a monodentate ligand due to the bulky chlorine atom being in direct proximity to one of the nitro­gen atoms (Wöhlert & Näther, 2013 ▸).

In this paper, we report on the crystal structure of [Fe^II^(C_4_H_3_ClN_2_)_2_(H_2_O)_4_](C_7_H_7_O_3_S)_2_ containing a cationic iron(II) complex with 2-chloro­pyrazine and aqua ligands, and tosyl­ate as an anion.

## Structural commentary   

**Figure 1:**
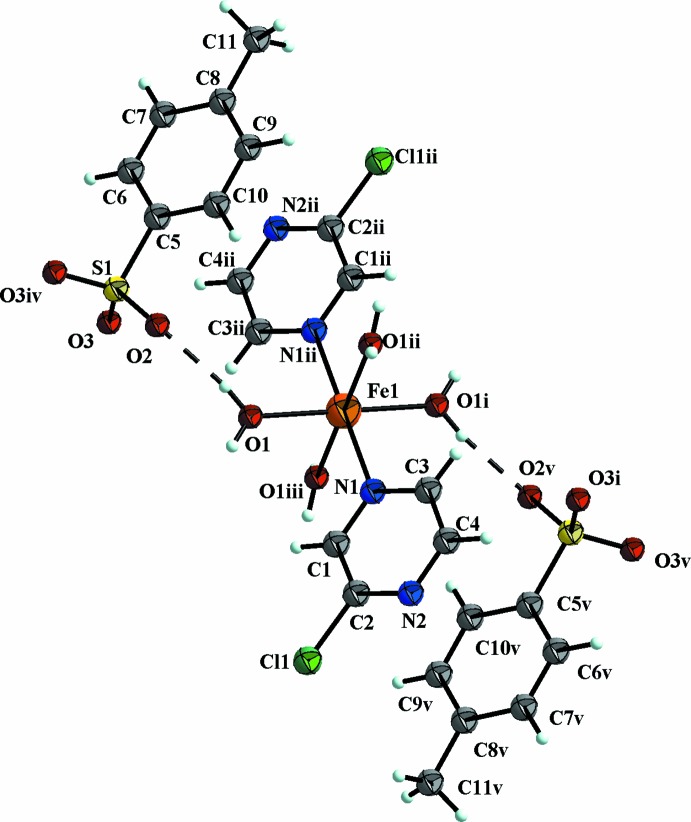
The structure of the cationic and anionic components in the title salt. Displacement ellipsoids are drawn at the 50% probability level. Hydrogen bonds are indicated by dashed lines. [Symmetry codes: (i) 1 − *x*, −*y*, 1 − *z*; (ii) 1 − *x*, *y*, 1 − *z*; (iii) *x*, −*y*, *z*; (iv) *x*, 1 − *y*, *z*; (v) 1 − *x*, −1 + *y*, 1 − *z*.]

The structure of the title compound consists of a complex cation [Fe(2-chloro­pyrazine)_2_(H_2_O)_4_]^2+^ and two tosyl­ate anions. The Fe^II^ atom, located on a special position with site symmetry 2/*m*, is sixfold coordinated by two N atoms of two symmetry-related 2-chloro­pyrazine ligands occupying the axial positions and four O atoms of four H_2_O mol­ecules forming the equatorial plane (Fig. 1 ▸). The distances between Fe^II^ and the O atoms [2.1004 (14) Å] of the H_2_O mol­ecules are significantly shorter than those between Fe^II^ and N [2.200 (2) Å] atoms of the two 2-chloro­pyrazine ligands, hence the resulting FeO_4_N_2_ octa­hedron is distorted. The metal-to-ligand distances clearly signalize the high-spin nature of the complex described in here (Shylin *et al.*, 2015 ▸). Similar structural features have been reported for other related compounds (Shylin *et al.*, 2013 ▸). The angles between the coordinating O atoms [O1^i^—Fe1—O1^iii^ = 90.83 (11)°; for symmetry codes see caption to Fig. 1 ▸], and coordinating N and O atoms [O1^ii^—Fe1—N1 = 90.68 (5)°] indicate only a small angular distortion.

## Supra­molecular features   

**Figure 2:**
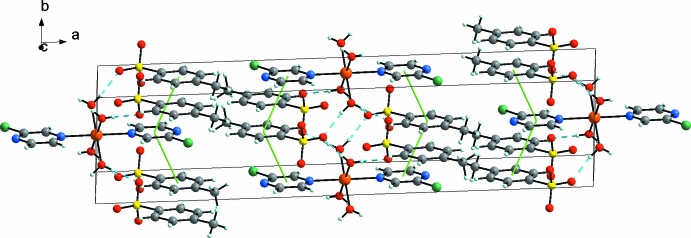
The crystal structure of the title compound, showing hydrogen bonds as dashed cyan lines and π–π contacts as green lines. Colour key: orange Fe, yellow S, blue N, grey C, green Cl, red O and white H.

In the title compound, the crystal packing is stabilized by O1—H1*A*⋯O2 and O1—H1*B*⋯O3^i^ hydrogen bonds (Table 1 ▸) between the complex cations and the counter-anions (Figs. 1 ▸ and 2 ▸). Only two O atoms of the tosyl­ate anion are involved in hydrogen bonding. Additional π –π stacking inter­actions (for numerical details, see: Table 2 ▸) between the pyrazine and benzene rings of the tosyl­ate anion contribute to the stabilization (Fritsky *et al.*, 2004 ▸) of the three-dimensional network (Fig. 2 ▸).

## Synthesis and crystallization   

Crystals of the title compound were obtained by adding 2-chloro­pyrazine (0.046 g, 0.4 mmol) to Fe(OTs)_2_·6H_2_O (0.096 g, 0.2 mmol) (OTs = *p*-toluene­sulfonate) and ascorbic acid (0.001 g) in water (5 ml). After seven days this yielded colourless blocks of the title compound that were collected, washed with water and dried in air. Yield 0.090 g (64%).

## Refinement   

Crystal data, data collection and structure refinement details are summarized in Table 3 ▸. All non-water H atoms were positioned geometrically and allowed to ride on their parent atoms, with *d*(C—H) = 0.95 Å for aromatic and 0.98 Å for CH_3_ hydrogen atoms. Because of the symmetry of the complete complex cation, methyl H atoms were modelled as equally disordered over two sets of sites. The H atoms of the water mol­ecule were located from a difference Fourier map and were modelled with a common isotropic displacement parameter fixed at 0.08 Å^2^. The O—H bonds lengths were constrained to 0.82 Å. The *U*_iso_ values were constrained to be 1.5*U*_eq_ of the carrier atom for methyl H atoms and 1.2*U*_eq_ for the remaining H atoms.

## Supplementary Material

Crystal structure: contains datablock(s) I. DOI: 10.1107/S2056989015010713/wm5168sup1.cif

Structure factors: contains datablock(s) I. DOI: 10.1107/S2056989015010713/wm5168Isup2.hkl

CCDC reference: 1404715

Additional supporting information:  crystallographic information; 3D view; checkCIF report

## Figures and Tables

**Table 1:** Hydrogen-bond geometry (, )

*D*H*A*	*D*H	H*A*	*D* *A*	*D*H*A*
O1H1*A*O2	0.82(2)	1.91(2)	2.7238(19)	171(4)
O1H1*B*O3^i^	0.81(2)	1.95(2)	2.7624(19)	177(3)

Symmetry code: (i) 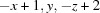.

**Table 2:** Geometric parameters of stacking (, )

centroid (2-chloropyrazine)centroid (tosylate anion)	3.7098(1)
centroid (2-chloropyrazine)centroid (tosylate anion)centroid (2-chloropyrazine)	130.283(1)

**Table 3:** Experimental details

Crystal data	
Chemical formula	[Fe(C_4_H_3_ClN_2_)_2_(H_2_O)_4_](C_7_H_7_O_3_S)_2_
*M*_r_	699.35
Crystal system, space group	Monoclinic, *C*2/*m*
Temperature (K)	133
*a*, *b*, *c* ()	30.691(3), 6.7321(3), 6.9435(6)
()	99.811(7)
*V* (^3^)	1413.63(19)
*Z*	2
Radiation type	Mo *K*
(mm^1^)	0.93
Crystal size (mm)	0.26 0.14 0.06

Data collection	
Diffractometer	Stoe IPDS II
Absorption correction	Numerical (*X-RED*; Stoe Cie, 2002 ▸)
*T*_min_, *T*_max_	0.697, 0.925
No. of measured, independent and observed [*I* > 2(*I*)] reflections	9102, 1630, 1380
*R*_int_	0.066
(sin /)_max_ (^1^)	0.633

Refinement	
*R*[*F* ^2^ > 2(*F* ^2^)], *wR*(*F* ^2^), *S*	0.028, 0.067, 1.00
No. of reflections	1630
No. of parameters	126
No. of restraints	2
H-atom treatment	H atoms treated by a mixture of independent and constrained refinement
_max_, _min_ (e ^3^)	0.38, 0.36

Computer programs: *X-AREA* and *X-RED* (Stoe Cie, 2002 ▸), *SHELXS97* (Sheldrick, 2008 ▸), *SHELXL2014* (Sheldrick, 2015 ▸), *OLEX2* (Dolomanov *et al.*, 2009 ▸) and *publCIF* (Westrip, 2010 ▸).

## supplementary crystallographic information

### Crystal data

**Table:**

[Fe(C_4_H_3_ClN_2_)_2_(H_2_O)_4_](C_7_H_7_O_3_S)_2_	*F*(000) = 720
*M**_r_* = 699.35	*D*_x_ = 1.643 Mg m^−^^3^
Monoclinic, *C*2/*m*	Mo *K*α radiation, λ = 0.71073 Å
*a* = 30.691 (3) Å	Cell parameters from 9102 reflections
*b* = 6.7321 (3) Å	θ = 1.4–26.7°
*c* = 6.9435 (6) Å	µ = 0.93 mm^−^^1^
β = 99.811 (7)°	*T* = 133 K
*V* = 1413.63 (19) Å^3^	Block, colourless
*Z* = 2	0.26 × 0.14 × 0.06 mm

### Data collection

**Table:**

Stoe IPDS II diffractometer	1380 reflections with *I* > 2σ(*I*)
φ scans and ω scans with κ offset	*R*_int_ = 0.066
Absorption correction: numerical (*X-RED*; Stoe & Cie, 2002)	θ_max_ = 26.7°, θ_min_ = 1.4°
*T*_min_ = 0.697, *T*_max_ = 0.925	*h* = −38→38
9102 measured reflections	*k* = −8→6
1630 independent reflections	*l* = −8→8

### Refinement

**Table:**

Refinement on *F*^2^	Primary atom site location: structure-invariant direct methods
Least-squares matrix: full	Hydrogen site location: mixed
*R*[*F*^2^ > 2σ(*F*^2^)] = 0.028	H atoms treated by a mixture of independent and constrained refinement
*wR*(*F*^2^) = 0.067	*w* = 1/[σ^2^(*F*_o_^2^) + (0.0395*P*)^2^] where *P* = (*F*_o_^2^ + 2*F*_c_^2^)/3
*S* = 1.00	(Δ/σ)_max_ = 0.001
1630 reflections	Δρ_max_ = 0.38 e Å^−^^3^
126 parameters	Δρ_min_ = −0.35 e Å^−^^3^
2 restraints	

### Special details

**Table:**

Geometry. All e.s.d.'s (except the e.s.d. in the dihedral angle between two l.s. planes) are estimated using the full covariance matrix. The cell e.s.d.'s are taken into account individually in the estimation of e.s.d.'s in distances, angles and torsion angles; correlations between e.s.d.'s in cell parameters are only used when they are defined by crystal symmetry. An approximate (isotropic) treatment of cell e.s.d.'s is used for estimating e.s.d.'s involving l.s. planes.

### Fractional atomic coordinates and isotropic or equivalent isotropic displacement parameters (Å^2^)

**Table:**

	*x*	*y*	*z*	*U*_iso_*/*U*_eq_	Occ. (<1)
Fe1	0.5000	0.0000	0.5000	0.01386 (13)	
Cl1	0.68495 (2)	0.0000	0.84806 (9)	0.02791 (16)	
O1	0.51119 (5)	0.2190 (3)	0.7183 (2)	0.0399 (4)	
N1	0.57111 (7)	0.0000	0.4870 (3)	0.0161 (4)	
N2	0.66150 (7)	0.0000	0.4683 (3)	0.0206 (4)	
C1	0.60191 (8)	0.0000	0.6491 (3)	0.0171 (5)	
H1	0.5932	0.0000	0.7741	0.021*	
C2	0.64619 (8)	0.0000	0.6350 (3)	0.0184 (5)	
C3	0.58590 (8)	0.0000	0.3161 (3)	0.0192 (5)	
H3	0.5652	0.0000	0.1977	0.023*	
C4	0.63061 (8)	0.0000	0.3081 (3)	0.0211 (5)	
H4	0.6396	0.0000	0.1839	0.025*	
S1	0.41620 (2)	0.5000	0.91435 (8)	0.01630 (14)	
O2	0.45581 (6)	0.5000	0.8231 (2)	0.0206 (4)	
O3	0.41275 (4)	0.32011 (19)	1.02718 (16)	0.0237 (3)	
C5	0.37118 (8)	0.5000	0.7189 (3)	0.0165 (5)	
C6	0.32788 (8)	0.5000	0.7567 (3)	0.0223 (5)	
H6	0.3226	0.5000	0.8876	0.027*	
C7	0.29303 (8)	0.5000	0.6040 (4)	0.0239 (5)	
H7	0.2637	0.5000	0.6308	0.029*	
C8	0.29970 (8)	0.5000	0.4102 (3)	0.0205 (5)	
C9	0.34283 (8)	0.5000	0.3749 (3)	0.0211 (5)	
H9	0.3480	0.5000	0.2438	0.025*	
C10	0.37849 (8)	0.5000	0.5263 (3)	0.0188 (5)	
H10	0.4078	0.5000	0.4993	0.023*	
C11	0.26090 (9)	0.5000	0.2452 (4)	0.0283 (6)	
H11A	0.2351	0.4424	0.2904	0.042*	0.5
H11B	0.2681	0.4209	0.1364	0.042*	0.5
H11C	0.2542	0.6367	0.2013	0.042*	0.5
H1A	0.4925 (10)	0.299 (5)	0.740 (5)	0.080*	
H1B	0.5338 (8)	0.251 (5)	0.791 (4)	0.080*	

### Atomic displacement parameters (Å^2^)

**Table:**

	*U*^11^	*U*^22^	*U*^33^	*U*^12^	*U*^13^	*U*^23^
Fe1	0.0130 (2)	0.0152 (2)	0.0129 (2)	0.000	0.00075 (16)	0.000
Cl1	0.0162 (3)	0.0456 (4)	0.0203 (3)	0.000	−0.0017 (2)	0.000
O1	0.0192 (7)	0.0481 (10)	0.0481 (9)	0.0068 (7)	−0.0063 (6)	−0.0343 (7)
N1	0.0165 (10)	0.0157 (10)	0.0159 (9)	0.000	0.0021 (7)	0.000
N2	0.0182 (10)	0.0241 (11)	0.0202 (9)	0.000	0.0051 (8)	0.000
C1	0.0183 (12)	0.0194 (12)	0.0139 (10)	0.000	0.0033 (9)	0.000
C2	0.0178 (12)	0.0194 (12)	0.0168 (10)	0.000	−0.0002 (9)	0.000
C3	0.0212 (13)	0.0210 (12)	0.0155 (10)	0.000	0.0032 (9)	0.000
C4	0.0216 (13)	0.0255 (13)	0.0170 (11)	0.000	0.0054 (9)	0.000
S1	0.0149 (3)	0.0189 (3)	0.0142 (3)	0.000	−0.0001 (2)	0.000
O2	0.0143 (8)	0.0235 (9)	0.0240 (8)	0.000	0.0030 (7)	0.000
O3	0.0224 (7)	0.0263 (7)	0.0203 (6)	−0.0031 (5)	−0.0025 (5)	0.0066 (5)
C5	0.0163 (12)	0.0173 (11)	0.0155 (10)	0.000	0.0014 (9)	0.000
C6	0.0190 (13)	0.0338 (15)	0.0139 (10)	0.000	0.0025 (9)	0.000
C7	0.0138 (12)	0.0354 (15)	0.0229 (12)	0.000	0.0040 (9)	0.000
C8	0.0183 (12)	0.0234 (13)	0.0181 (11)	0.000	−0.0018 (9)	0.000
C9	0.0211 (13)	0.0268 (13)	0.0153 (11)	0.000	0.0028 (9)	0.000
C10	0.0176 (12)	0.0223 (12)	0.0171 (10)	0.000	0.0046 (9)	0.000
C11	0.0217 (13)	0.0391 (16)	0.0216 (12)	0.000	−0.0030 (10)	0.000

### Geometric parameters (Å, º)

**Table:**

Fe1—O1^i^	2.1004 (14)	S1—O2	1.4632 (17)
Fe1—O1^ii^	2.1004 (14)	S1—O3^iv^	1.4560 (12)
Fe1—O1^iii^	2.1004 (14)	S1—O3	1.4560 (12)
Fe1—O1	2.1004 (14)	S1—C5	1.764 (2)
Fe1—N1^i^	2.200 (2)	C5—C6	1.398 (3)
Fe1—N1	2.200 (2)	C5—C10	1.393 (3)
Cl1—C2	1.733 (2)	C6—H6	0.9500
O1—H1A	0.820 (18)	C6—C7	1.372 (3)
O1—H1B	0.814 (18)	C7—H7	0.9500
N1—C1	1.341 (3)	C7—C8	1.395 (3)
N1—C3	1.341 (3)	C8—C9	1.387 (3)
N2—C2	1.321 (3)	C8—C11	1.506 (3)
N2—C4	1.333 (3)	C9—H9	0.9500
C1—H1	0.9500	C9—C10	1.383 (3)
C1—C2	1.379 (3)	C10—H10	0.9500
C3—H3	0.9500	C11—H11A	0.9800
C3—C4	1.383 (4)	C11—H11B	0.9800
C4—H4	0.9500	C11—H11C	0.9800
			
O1^i^—Fe1—O1^iii^	90.83 (11)	N2—C4—H4	118.8
O1^i^—Fe1—O1	180.0	C3—C4—H4	118.8
O1^iii^—Fe1—O1	89.17 (11)	O2—S1—C5	105.44 (10)
O1^iii^—Fe1—O1^ii^	180.0	O3^iv^—S1—O2	112.02 (6)
O1^i^—Fe1—O1^ii^	89.17 (11)	O3—S1—O2	112.02 (6)
O1^ii^—Fe1—O1	90.83 (11)	O3—S1—O3^iv^	112.56 (10)
O1^iii^—Fe1—N1	89.32 (5)	O3—S1—C5	107.14 (7)
O1^ii^—Fe1—N1^i^	89.32 (5)	O3^iv^—S1—C5	107.14 (7)
O1^i^—Fe1—N1^i^	89.32 (5)	C6—C5—S1	120.03 (17)
O1^ii^—Fe1—N1	90.68 (5)	C10—C5—S1	120.37 (18)
O1—Fe1—N1	89.32 (5)	C10—C5—C6	119.6 (2)
O1^i^—Fe1—N1	90.68 (5)	C5—C6—H6	120.1
O1^iii^—Fe1—N1^i^	90.68 (5)	C7—C6—C5	119.7 (2)
O1—Fe1—N1^i^	90.68 (5)	C7—C6—H6	120.1
N1^i^—Fe1—N1	180.0	C6—C7—H7	119.3
Fe1—O1—H1A	124 (3)	C6—C7—C8	121.5 (2)
Fe1—O1—H1B	131 (2)	C8—C7—H7	119.3
H1A—O1—H1B	105 (3)	C7—C8—C11	120.5 (2)
C1—N1—Fe1	121.88 (15)	C9—C8—C7	118.2 (2)
C1—N1—C3	116.5 (2)	C9—C8—C11	121.4 (2)
C3—N1—Fe1	121.60 (16)	C8—C9—H9	119.3
C2—N2—C4	115.0 (2)	C10—C9—C8	121.4 (2)
N1—C1—H1	119.9	C10—C9—H9	119.3
N1—C1—C2	120.2 (2)	C5—C10—H10	120.2
C2—C1—H1	119.9	C9—C10—C5	119.6 (2)
N2—C2—Cl1	116.93 (19)	C9—C10—H10	120.2
N2—C2—C1	124.4 (2)	C8—C11—H11A	109.5
C1—C2—Cl1	118.71 (18)	C8—C11—H11B	109.5
N1—C3—H3	119.2	C8—C11—H11C	109.5
N1—C3—C4	121.6 (2)	H11A—C11—H11B	109.5
C4—C3—H3	119.2	H11A—C11—H11C	109.5
N2—C4—C3	122.4 (2)	H11B—C11—H11C	109.5
			
Fe1—N1—C1—C2	180.000 (1)	O2—S1—C5—C10	0.000 (1)
Fe1—N1—C3—C4	180.000 (1)	O3^iv^—S1—C5—C6	−60.51 (6)
N1—C1—C2—Cl1	180.000 (1)	O3—S1—C5—C6	60.51 (6)
N1—C1—C2—N2	0.000 (1)	O3^iv^—S1—C5—C10	119.49 (6)
N1—C3—C4—N2	0.000 (1)	O3—S1—C5—C10	−119.49 (6)
C1—N1—C3—C4	0.000 (1)	C5—C6—C7—C8	0.000 (1)
C2—N2—C4—C3	0.000 (1)	C6—C5—C10—C9	0.000 (1)
C3—N1—C1—C2	0.000 (1)	C6—C7—C8—C9	0.000 (1)
C4—N2—C2—Cl1	180.000 (1)	C6—C7—C8—C11	180.000 (1)
C4—N2—C2—C1	0.000 (1)	C7—C8—C9—C10	0.000 (1)
S1—C5—C6—C7	180.000 (1)	C8—C9—C10—C5	0.000 (1)
S1—C5—C10—C9	180.000 (1)	C10—C5—C6—C7	0.000 (1)
O2—S1—C5—C6	180.000 (1)	C11—C8—C9—C10	180.000 (1)

Symmetry codes: (i) −*x*+1, −*y*, −*z*+1; (ii) −*x*+1, *y*, −*z*+1; (iii) *x*, −*y*, *z*; (iv) *x*, −*y*+1, *z*.

### Hydrogen-bond geometry (Å, º)

**Table:**

*D*—H···*A*	*D*—H	H···*A*	*D*···*A*	*D*—H···*A*
O1—H1*A*···O2	0.82 (2)	1.91 (2)	2.7238 (19)	171 (4)
O1—H1*B*···O3^v^	0.81 (2)	1.95 (2)	2.7624 (19)	177 (3)

Symmetry code: (v) −*x*+1, *y*, −*z*+2.

## References

[bb1] Dolomanov, O. V., Bourhis, L. J., Gildea, R. J., Howard, J. A. K. & Puschmann, H. (2009). *J. Appl. Cryst.* **42**, 339–341.

[bb2] Fritsky, I. O., Świątek-Kozłowska, J., Dobosz, A., Sliva, T. Y. & Dudarenko, N. M. (2004). *Inorg. Chim. Acta*, **357**, 3746–3752.

[bb3] Gural’skiy, I. A., Quintero, C. M., Abdul-Kader, K., Lopes, M., Bartual-Murgui, C., Salmon, L., Zhao, P., Molnar, G., Astruc, D. & Bousseksou, A. (2012). *J. Nanophoton.* **6**, 063517.

[bb4] Gütlich, P. & Goodwin, H. (2004). *Spin Crossover in Transition Metal Compounds I*, pp. 1–6. Berlin, Heidelberg: Springer-Verlag.

[bb5] Sheldrick, G. M. (2008). *Acta Cryst.* A**64**, 112–122. 10.1107/S0108767307043930 18156677

[bb6] Sheldrick, G. M. (2015). *Acta Cryst.* C**71**, 3–8.

[bb7] Shylin, S. I., Gural’skiy, I. A., Bykov, D., Demeshko, S., Dechert, S., Meyer, F., Hauka, M. & Fritsky, I. O. (2015). *Polyhedron*, **87**, 147–155.

[bb8] Shylin, S. I., Gural’skiy, I. A., Haukka, M. & Golenya, I. A. (2013). *Acta Cryst.* E**69**, m280. 10.1107/S1600536813010362 PMC3647816 23723782

[bb9] Stoe & Cie (2002). *X-RED* and *X-AREA*. Stoe & Cie, Darmstadt, Germany.

[bb10] Westrip, S. P. (2010). *J. Appl. Cryst.* **43**, 920–925.

[bb11] Wöhlert, S. & Näther, C. (2013). *Inorg. Chim. Acta*, **406**, 196–204.

[bb12] Wriedt, M. & Näther, C. (2011). *Z. Anorg. Allg. Chem.* **637**, 666–671.

